# Treatment of Catarrh of the Antrum

**Published:** 1882-06

**Authors:** 


### TREATMENT OF CATARRH OF THE ANTRUM.

Dr. G. Wolfram reports in *Le Progrès Dentaire* the following case: The patient was a man of fifty-five years, of fine appearance, and had always been in good health, save occasional attacks of influenza, and of nasal catarrh during winter seasons. In April of 1876 he complained of pain in left superior maxillary, radiating toward orbital region, also the frontal. He thought that the first superior left molar caused, or was the seat of the pain, and had it extracted, but found the tooth sound and roots healthy. In the afternoon of the same day he found a disagreeable taste, also an offensive odor, which was proven to arise from the nose.

Some relief was afforded him by a physician who ordered a nasal douche of the solutions of salicylic acid and of chlorate of potash, but his condition was only ameliorated.

Dr. Wolfram diagnosed, in addition to the nasal catarrh, that there was also disease of the maxillary sinus and that the true origin of the offensive pus was there. As he could not reach the antrum by means of the douche, he forced through the nose, by means of a small inhaling apparatus, a solution of glycerine with two per cent of tannin ; afterward he substituted a solution of acetate of alumina, from six-tenths to one per cent. A cure was effected in six weeks, with no return of symptoms for two years.

